# Top-Down Auditory Plasticity: Acceptable Noise Level Predicts and Reflects the Effect of Perceptual Learning in Experience-Induced Plasticity

**Published:** 2019

**Authors:** Elahe Ravanshenas, Hamid Jalilvand, Alireza Akbarzade Baghban

**Affiliations:** 1 1.Department of Audiology, School of Rehabilitation, Shahid Beheshti University of Medical Sciences, Tehran, Iran; 2 School of Rehabilitation, Shahid Beheshti University of Medical Sciences, Tehran, Iran

**Keywords:** Acceptable noise level, Noise; Hearing, Auditory efferent system

## Abstract

**Objective:**

In the auditory system, tinnitus and superior speech perception in noise are examples of negative and positive plasticity that can result from sensory neural hearing loss and life experiences dealing with more complex stimuli and learning, respectively. The main objective of this study was to determine the relationship between acceptable noise level (ANL) values and perceptual learning in individuals exposed to unavoidable occupational noise.

**Materials & Methods:**

Here we document a form of plasticity in top-down auditory pathways through the measurement of the acceptable noise level in 60 adults, 27 females and 33 males, with normal hearing (Amiraalam state Hospital, Tehran, Iran 2016). Individuals were assigned to one of two groups: those with and without the occupational experience of speech perception in noise.

**Results:**

The test group had statistically significant lower acceptable noise level and significantly higher background noise level scores compared with the control group.

**Conclusion:**

Using acceptable noise level, we attributed differences in individuals’ abilities to tolerate varying amounts of background noise and speech perception in noise function to the auditory efferent system. Working in crowded locations due to job nature can influence differences in speech perception in noise function.

## Introduction

Speech perception in noise is one of the most complex mental activities encountered in everyday life and is dependent upon the optimal functioning of peripheral hearing, central auditory processing and cognition, and individual daily experiences (1). The auditory cortex is organized through the experiences of environmental sounds (2), and these experiences influence the development of differences among people in the ability to process auditory sounds (3). 

Different human experiences and lifestyles can influence the abilities of individuals to perceive speech in noise. Speech perception and the short- and long-term experiences that influence speech perception have been shown to influence auditory nervous processing via click-evoked auditory brainstem responses (cABR) that originate from the brainstem (1). For example, the experience of playing music and being bilingual are two examples of perceptual learning that influence speech perception in noise and manifest in the form of physiological differences and neuroplasticity-based on learning, which influences properties of neural decoding, thus resulting in improved speech perception in noise (1).

Through evolutionary time, the brainstem has been modified by human experience and learning; in musicians, changes in the structure and function of the brain occur as a result of learning and experiencing different sounds. While learning, the brain works as an integrated unit in which the auditory efferent system is associated with non-classical auditory regions in the brain that produce basic changes in the cortical and subcortical response properties in the classical auditory system. Learning can result in subcortical physiological changes via top-down mechanisms. Learning plays an important role in the plasticity of the auditory efferent system (3).

Studies conducted on auditory perceptual learning support the notion that intensive auditory training leads to long-term neurological changes in the auditory cortex of adults in humans and other animals (4). The effect of learning on speech perception was demonstrated in noise. For example, learning a second language and playing music are ways in which people can improve speech perception in noise. This learning affects the properties of neural decoding via physiological changes and learning-dependent neuroplasticity in ascending auditory pathways; therefore, learning a second language and playing music can enhance the individual capability of speech perception in noise.

Perceptual learning in adults can be strongly attributed to one’s level of attention, physical and mental activity, and social situations (1, 3, 4). The effects of these factors have been researched in the plasticity of central auditory processing, which results in improved speech perception capabilities under noisy conditions. In other words, the ability of the auditory efferent system to suppress undesirable auditory inputs is an important effect that has an active role in signal processing and modulating auditory input stimulants (4). 

In the corticofugal system, the descending pathway extends from the auditory cortex to the subcortical regions and nucleus. The descending pathway branches from the brainstem to the inner ear via efferent fibers of the medial olivocochlear bundle (MOCB), ending at the cochlea where the efferent fibers make contact with the outer hair cells, thus modulating active amplification mechanisms (4, 5). The function of the MOCB is to reduce the number of reflexive responses to sounds (5), suppress basilar membrane response (6), and consequently, protect the ear against extremely loud sounds (5). In addition, the MOCB facilitates speech perception in noise and improves signal-to-noise ratio and selective attention (5, 7-9). This ability contributes to the enhancement of the signal-to-noise ratio through the reduction of the auditory nerve response to noise input and an increase in the auditory nerve response to transient sounds (6, 7, 10). A superior ability of some individuals was shown to extract signals from noise. This phenomenon is derived from forceful perceptual learning (4). We assumed that individuals with long-term experience of speech perception in noise conform to this fact.

One of the tests in the field of speech-in-noise is the acceptable noise level (ANL) test, and its ability to assess the auditory efferent system has been examined. The ANL test can reveal the function of the ability of the auditory efferent system to process signals in noise (11, 12). The ANL test was introduced as a procedure for defining acceptable noise levels while people listen to a speech (13). The ANL test measures the highest level of background noise that a person can tolerate while listening to a running speech (14, 15). To measure ANL, a running speech is first presented to a person and the person’s most comfortable level (MCL) for speech is measured. Background noise (typically babble noise) is then added and increased until the person indicates he/she cannot tolerate any additional noise while still listening to the running speech without any tension or fatigue. This tolerable level of noise is called the background noise level (BNL). BNL is then subtracted from MCL to yield the ANL (ANL = MCL ‒ BNL). Smaller ANL scores (≤7 dB) indicate that listeners can accept higher levels of background noise while listening to the speech, whereas larger ANL scores (≥13 dB) indicate the opposite (12, 14, 15).

ANL through time is partly stable and reliable. Additionally, ANL scores are not correlated to age, hearing sensitivity, sex, background noise, personal preference for background noise, spectral shape of environmental noise, reverberation time, primary language of the listener, middle ear function, acoustic reflex thresholds or contralateral suppression of otoacoustic emissions (OAE), or speech understanding in noise scores. Regardless of simplicity and ease in performing, ANL scores provide an indication of the capabilities of the auditory efferent system in suppressing noise (11, 12, 15-18). The ANL test registers in the central nervous system and the upper portion of the olivocochlear complex (11, 12).

The main objective of this study was to determine the relationship between ANL values and perceptual learning in individuals exposed to unavoidable occupational noise. We asked if there would be any differences between ANL scores in people who work in noisy environments and those who do not.

## Materials & Methods

**Participants**

Sixty individuals with normal hearing (27 females and 33 males) were enrolled. The participants were placed into one of two groups: Group 1 (test group) had occupational experience of speech perception in noise; Group 2 (control group) did not have this occupational experience. Study participants ranged in age between 23 and 48 yr old. 

First, an otoscopy examination was performed to ensure each participant had a healthy and intact tympanic membrane. Then, the pure tone audiometry in range of octave frequencies (250-8000 Hz) was performed using an AC33 Interacoustic audiometer (Interacoustic Co., Denmark). To be included in the study in either group, participants were required to have normal hearing thresholds (≤25 dB HL at all octave frequencies), non-existence or lack of any otologic impairments and neurological or cognitive deficits, and to have a non-musical background. Additionally, Group 1 participants were required to have worked at least 1 year in a noisy environment (e.g., cashiers who work in crowded, busy places, such as state hospitals). We selected Amiraalam state Hospital, Tehran, Iran (2016), Iran as a busy place and Rehabilitation College at the Shahid Beheshti University of Medical Science as a quiet place. Participants of Group 1 were employees of Amiraalam Hospital in Tehran, whereas Group 2 participants were employees and students of the Rehabilitation College at the Shahid Beheshti University of Medical Science. 

All methods were carried out in accordance with relevant guidelines and regulations of Audiology Department of Shahid Beheshti University of Medical Sciences. All experimental protocols of this study were approved by the Ethics Committee of Rehabilitation School of Shahid Beheshti University of Medical Sciences. Before participating in the study, informed consent was obtained from all subject. 

**ANL Measurement**

A loudspeaker was placed 1 m in front of each individual at 0° Azimuth). The Persian version of the ANL test was used in this study; this version has been used in a previous study (19-21). A running speech with a female voice telling a story was used. The noise of 12 people babbling was used as the test noise (19-21). The test was adequately explained to each participant before beginning. 

ANL score is obtained in three steps: Firstly, a running speech is heard from a loudspeaker as it rises and falls; this yields the person’s MCL. Babble noise is then added to the speech and is increased until the person can no longer tolerate it without any annoyance or discomfort. Lastly, the ANL score is obtained by subtracting MCL from BNL: ANL = MCL ‒ BNL.

**Statistical Method**

The Kolmogorov–Smirnov test was used for assessing the normal distribution of data. Pearson’s correlation was used to assess the relationships among variables, and the independent t-test was used to compare data between groups. All analyses were performed using SPSS version 16 (ver.16.0. Chicago, IL, USA).

## Results

There were no statistically significant differences between the ages of the groups (Table 1).

**Table 1:** Mean ages of both groups

	Groups	Mean ± SD	Range
Age	G1 (Test)	34.80 ± 6.93	23 ‒ 48
	G2 (Control)	34.97 ± 9.20	23 ‒ 55

Group 1 participants had worked an average of 10.65 ± 7.93 yr in noisy places (range =1-28 yr).

**Figure 1:**
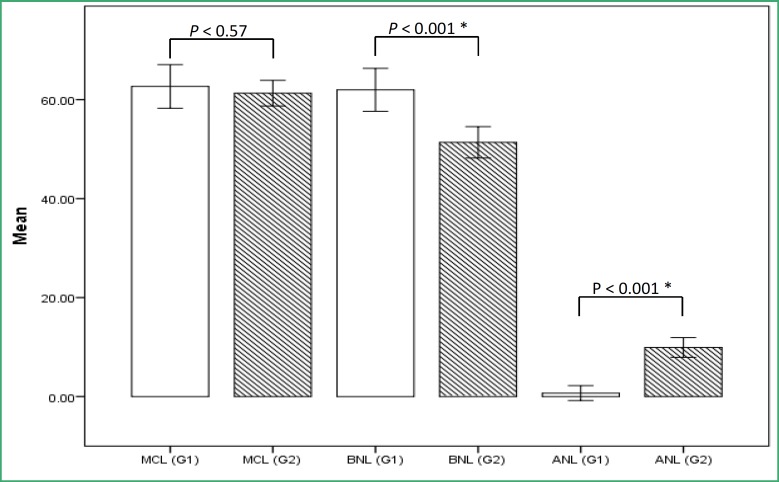
Mean and standard errors of MCL, BNL, and ANL scores in both groups (Group 1 (cashiers) and Group 2 (control)

The distribution of data for the ANL, MCL, and BNL variables was normal in both groups (*P*<0.05). There was a statistically significant difference (*P*<0.01) only between the ANL and BNL scores in both groups. The ANL score of Group 1 was significantly lower than that of Group 2 (0.7±4.06 vs. 9.9±5.34 dB,* P*<0.001). The BNL of Group 1 was significantly higher than that of Group 2 (61.9±11.62 vs. 51.3±8.49 dB, *P*<0.001, Figure 1). No significant difference between the MCLs of both groups was observed (62.6±11.79 vs. 61.3±6.94 dB, *P*=0.59, Figure 1).

There was a slight inverse relationship between BNL and ANL scores in Group 2 only (r=−0.579, P=0.000). There was a significantly strong relationship between years of experience and ANL scores (r = 0.95, P=0.000).

## Discussion

Our data demonstrate that ANL in people who work in noisy environments is superior to those who do not; we attribute this to experience-induced perceptual learning. Auditory learning is a product of top-down circuits in the auditory system, and it appears learning plays an important role in the plasticity of auditory descending pathways (3). The adult auditory cortex is as a dynamic and adaptive processing center (4), whereas visual, somatosensory, limbic, and associated regions are non-auditory regions that may weaken auditory system function. Memory, attention, communication skills, and learning can improve auditory system function (3). For example, experience-induced learning and can modify one’s auditory processing centers. Modification of the brain structure and function has been described in musicians, individuals who experience different sounds, via cABR (3). Auditory processing abilities in individuals differ based on varying experiences of sound. cABR is a brainstem response to complex stimuli that presents the precise measurement of sound processing and is deeply influenced by cognition factors like education and communication skills. cABR is influenced by aging, reading ability, cognitive ability, experience in playing music, and bilingualism. Thus, cABR is a plasticity indicator of the auditory system. cABR has a fine relationship between the decoding of F0 in the brainstem and hearing ability in noise (3). cABR is one measurement that reflects the evidence of plasticity in neural changes and decoding centers (ascending pathways) in the auditory system (4). Therefore, auditory training, perceptual learning, and experiences with sound are skills that influence the plasticity of both ascending and descending central auditory processing (4). Interestingly, the auditory efferent system (descending pathways) has the same importance as the afferent system involved in signal processing and shaping neural representation (4){de Boer, 2008 #4;de Boer, 2008 #4}. Thus, just as learning-dependent plasticity occurs in ascending pathways (4), this study demonstrated evidence of plasticity in descending pathways, which during experiences with sound resulted in improved ability in suppressing undesirable sounds in adults with normal hearing. 

After the discovery of efferent system by Grant Rasmussen, many studies have been conducted in an attempt to understand how the efferent system works (3, 8). Improved hearing perception in noise function is an auditory efferent activity. For example, individuals with stronger olivocochlear reflex or stronger OAE suppression show improved speech understanding in noise, and there is a basic relationship between distortion product OAE suppression amplitude and speech-in-noise scores (5-7, 9, 10).

Deficiencies in contralateral suppression have been observed in the impaired olivocochlear bundle (OCB) of the auditory efferent system as a result of aging (6), presences of Type II Diabetes Mellitus (9), and vestibular neurectomy (9). These deficiencies lead to a degradation of speech perception in noise. Moreover, MOCB deficiency has been shown to impair the ability of monkeys to discern vowels in noise, although not under silent conditions (6). Crossed OCB in patients resulting in decreased attention is observed in normal individuals without any audiological impairment. In this effect, individuals with normal hearing capabilities show increased thresholds for stimulus tones in unexpected frequencies, but the opposite effect is seen in the patient group with audiological impairments (6). 

In individuals with perfect hearing, efferent fibers suppress the basilar membrane responses in the region of the cochlea that is most sensitive to low frequencies (6). The amount of auditory  efferent activity is proportional to ambient noise level (6). Noise stimulation decreases the range of the signal that can produce a response in the auditory nerve (reduction of dynamic range) via an increase in the basic firing rate and a decrease in the firing rate wherein has been saturated fibers (5). In addition, OCB electrical or acoustical stimulation has been shown to increase one’s compound action potential response to tone in the presence of noise. Its mechanism is complex, but it likely involved some form of adaptation. During long-term exposure to background noise, the auditory nerve response adapts and becomes less responsive to new sounds. The auditory efferent system decreases a person’s response to continuous noise and increases their response to new sounds presented as part of the background noise (6-8). This occurs via reducing adaptation, particularly when the noise is continuous and the signal is transient; this constitutes an antimasking role in speech comprehension in the MOCB (6, 8, 9).

Using the ANL test, this study demonstrates the role of the auditory efferent system in noisy environments. The efficiency of the ANL test has been confirmed by previous studies (11, 12). Thus, here we proceed on documenting the importance of the role of human experiences in the functioning of the auditory efferent system. The long-term exposure of some individuals to noisy and crowded circumstances may be inevitable depending on one’s occupation. Noisy situations influence the plasticity of the auditory efferent system and may be viewed as one style of learning. 

Changes in the auditory efferent system can be documented using the ANL test. Here, the participants, experienced long-term exposure to noisy environments would have a stronger or more optimal auditory efferent system than those who did not, and consequently, have lower ANL scores. A low ANL score indicates that a person can tolerate more background noise, whereas those with high ANL scores can tolerate less background noise while listening to a story-like speech (12, 15). The ANL test originates from the upper part of the olivocochlear complex in the central auditory processing system (11, 12). Researchers have used this tool to exhibit differences in individuals’ abilities to tolerate different amounts of noise. While the ANL test is independent of many factors (15-18), language and babble noise construction and cultural background influence the score of the ANL test (18). In this study, for the first time, an individual’s occupation can influence the ANL test. Furthermore, we demonstrated the role of experience-induced learning in the plasticity of the auditory top-down pathways. We used the ANL test to show differences in auditory efferent system function in individuals from two different groups: individuals with and without the occupational experience of speech perception in noise. Our findings demonstrate that job conditions can affect ANL scores. This result must be considered when consulting adults with normal hearing that complain of a failure in speech comprehension under normal conditions and when deciding on an appropriate hearing-aid device for patients with hearing loss by measuring ANL score in clinical practice.

Measurement of the amount of environmental noise is recommended in future studies for more comparison of occupational conditions in both groups. 

**In conclusion,** this research introduces the importance of human experiences and lifestyles as one of the causes of discrepancies in some individuals in the more easy hearing of undesirable sounds and their more optimal function in noisy conditions. Our results showed symptoms of neural plasticity (towards the upper MOCB level) in the top-down pathways of auditory processing in adults with normal hearing that relate to perceptual learning.

## References

[1] Anderson S, White-Schwoch T, Parbery-Clark A, Kraus N (2013). A dynamic auditory-cognitive system supports speech-in-noise perception in older adults. Hear Res.

[2] White EJ, Hutka SA, Williams LJ, Moreno S (2013). Learning, neural plasticity and sensitive periods: implications for language acquisition, music training and transfer across the lifespan. Front Syst Neurosci.

[3] Guinan JJ JR, Popper AN, Fay RR (2014). Cochlear mechanics, otoacoustic emissions, and medial olivocochlear efferents: twenty years of advances and controversies along with areas ripe for new work. Perspectives on Auditory Research.

[4] de Boer J, Thornton AR (2008). Neural correlates of perceptual learning in the auditory brainstem: efferent activity predicts and reflects improvement at a speech-in-noise discrimination task. J Neurosci.

[5] de Boer J, Thornton AR, Krumbholz K (2012). What is the role of the medial olivocochlear system in speech-in-noise processing?. J Neurophysiol.

[6] Brown GJ, Ferry RT, Meddis R (2010). A computer model of auditory efferent suppression: implications for the recognition of speech in noise. J Acoust Soc Am.

[7] Andéol G, Guillaume A, Micheyl C, Savel S, Pellieux L, Moulin A (2011). Auditory efferents facilitate sound localization in noise in humans. J Neurosci.

[8] Ciuman RR (2010). The efferent system or olivocochlear function bundle - fine regulator and protector of hearing perception. Int J Biomed Sci.

[9] Prabhu P, Shanthala SP (2016). Efferent Auditory System Functioning and Speech Perception in Noise in Individuals with Type II Diabetes Mellitus. Journal of Phonetics & Audiology.

[10] Guinan JJ JR (2006). Olivocochlear efferents: anatomy, physiology, function, and the measurement of efferent effects in humans. Ear Hear.

[11] Harkrider AW, Tampas JW (2006). Differences in responses from the cochleae and central nervous systems of females with low versus high acceptable noise levels. J Am Acad Audiol.

[12] Shetty HN, Mahadev S, Veeresh D (2014). The Relationship Between Acceptable Noise Level and Electrophysiologic Auditory Brainstem and Cortical Signal to Noise Ratios. Audiol Res.

[13] Nabelek AK, Tucker FM, Letowski TR (1991). Toleration of background noises: relationship with patterns of hearing aid use by elderly persons. J Speech Hear Res.

[14] Shetty HN, Subbanna S (2015). Acceptable noise level as a deciding factor for prescribing hearing aids for older adults with cochlear hearing loss – A scoping review. J Otol.

[15] Freyaldenhoven MC Acceptable Noise Level (ANL): Research and Current Application.

[16] Freyaldenhoven MC, Smiley DF, Muenchen RA, Konrad TN (2006). Acceptable noise level: reliability measures and comparison to preference for background sounds. J Am Acad Audiol.

[17] Kim JH, Lee JH, Lee HK (2014). Advantages of Binaural Amplification to Acceptable Noise Level of Directional Hearing Aid Users. Clin Exp Otorhinolaryngol.

[18] Shi LF, Azcona G, Buten L (2015). Acceptance noise level: effects of the speech signal, babble, and listener language. J Speech Lang Hear Res.

[19] Ahmadi A, Fatahi J, Keshani A, Jalilvand H, Modarresi Y, Jalaie Sh (2015). Developing and evaluating the reliability of acceptable noise level test in Persian language. Sci J Rehabil Med.

[20] Aghsoleimani M, Jalilvand H, Mahdavi ME, Nazeri AR, Kamali M (2018). The acceptable noise level benefit from directionality for listeners with severe hearing loss. Clin Exp Otorhinolaryngol.

[21] Ahmadi R, Jalilvand H, Mahdavi ME, Ahmadi A, Akbarzade Baghban AR (2018). The effect of hearing aid digital noise reduction and directionality on acceptable noise level. Clin Exp Otorhinolaryngol.

